# An Approach to Dynamic Sensing Data Fusion

**DOI:** 10.3390/s19173668

**Published:** 2019-08-23

**Authors:** Yunfei Yin, Liufa Guan, Chengen Zheng

**Affiliations:** 1 College of Computer Science, Chongqing University, Chongqing 400044, China; 2 Key Lab. of Dependable Service Computing in Cyber Physical Society of Ministry of Education, Chongqing 400044, China

**Keywords:** dynamic acquisition, feedback control, sensor device, sensing data

## Abstract

For the research and development of sensor systems, the collection and fusion of sensing data is the core. In order to make sensor data acquisition change with the change in environment, a dynamic data acquisition and fusion method based on feedback control is proposed in this paper. According to the sensing data acquisition and fusion model, the optimal acquisition of sensor data is achieved through real-time dynamic judgment of the collected data, decision-making of the next acquisition time interval, and adjustment. This model enables the sensor system to adapt to different environments. An experimental study of the proposed model was carried out on an experimental platform, and the results show that the proposed model can not only reflect the change in sensing data but also improve the transmission efficiency.

## 1. Introduction

Sensors are one of the main equipment for data acquisition and an important bridge to obtain environmental information. How to process the data collected by sensors is a hot research topic in industry. Because of the complex and changeable environment, sensors cannot perform their best. How to dynamically adjust the sensor acquisition frequency according to environmental changes and then fuse the sensor data is therefore very meaningful.

Sensor data acquisition indicators include accuracy and bandwidth. The former requires the collected data to accurately reflect the objective facts, while the latter requires the collected data to be as few as possible in order to achieve the fastest transmission efficiency. At present, there are two kinds of sensor data acquisition methods in industry and academia: the best accuracy method and the minimum bandwidth method.

In order to improve the accuracy of the best accuracy method, we usually adopt the method of shortening the acquisition time interval. The advantages of this method are high precision and accurate prediction, while the disadvantages are larger transmission bandwidth occupation and lower handling efficiency. The sensor data collection of DSP chip TMS320VC33 [1] and the sensor data collection based on the Advantech card PCI-1811L [2] are examples of the best accuracy method.

In the minimum bandwidth method, in order to reduce the number of acquisitions, we usually expand the acquisition time interval. Therefore, the minimum bandwidth method reduces the number of acquisitions and achieves the real-time processing requirements for environmental data. The advantage of this method is high collection efficiency that is suitable for concurrency and real-time systems, while the disadvantage is lower collection precision. The multiplex signal asynchronous sampling model proposed by Shi et al. [3], microbial environmental data collection [4], data collection in biological and environmental sciences [5], and collection of experimental data for aerosol monitoring cyclones [6] belong to this sensor data acquisition method.

In practical applications, the best accuracy method and the minimum bandwidth method are often combined. The merged method is a compromise method, which uses as little bandwidth as possible to meet the basic acquisition accuracy, thus promoting the progress of environmental data acquisition [7,8,9].

However, the traditional method is a static method, which does not take into account the dynamic characteristics of current environmental data. If we can mine the changes in sensor data from the current collected data in the recent period, then we can regard them as a priori knowledge to dynamically adjust the collection intervals and therefore achieve collection precision and maximum utilization of transmission bandwidth. Based on this, we adopted a dynamic collection method for sensor data that predicts the changing degree of data according to the collected sensor data and decides whether the current acquisition timeintervals need to be dynamically adjusted. If the timeintervals need to be adjusted, then the algorithm will be immediately conducted; otherwise, the original values are maintained. The method proposed in this paper can achieve collection precision and maximum utilization of transmission bandwidth.

In this paper, we propose a dynamic and adaptive data acquisition interval adjustment method to solve the problem of data acquisition efficiency and accuracy.

The key issues are judgment of data changing degree and dynamic regulation of acquisition timeintervals. The judgment process of data changing degree is as follows. First, collect N data and compute the predicted value of the N+1 data based on the collected N data. Second, collect the N+1 data and compute its measured value. Finally, compare the predicted value and the measured value of the N+1 data.If the difference between the predicted value and the measured value of the N+1 data exceeds the designated threshold, the acquisition timeintervals need to be adjusted. The existing research includes the Delta model [10], Markov model [11], Shirai model [12], and data detection model [13]. The process of dynamically adjusting the acquisition timeintervals is as follows. According to the current changing degree of collection data, adaptively adjust the acquisition timeintervals, i.e., designate a basic acquisition time unit in advance, and the practical collection timeinterval is K times the basic acquisition time unit (K is an integer).When the collection timeinterval needs to be increased, appropriately increase the value of K; otherwise appropriately decrease the value of K acquisition time. The existing research includes [14,15,16].

In order to verify the method of dynamic sensing data acquisition, we used the W5 experimental system [17], which has common sensor types. This is an experimental system for monitoring, image acquisition, data analysis, meteorological prediction, and pesticide spraying. It is simple and direct and was therefore chosen as the experimental platform in this study.

The quality and efficiency of sensor data acquisition are of great significance to Internet of Things equipment, namely, (1) improving the quality of Internet of Things equipment because accurate sensor data is the scientific basis for improving the quality of equipment; (2) real-time understanding of the status of equipment because staff can understand the status of the system in real time through sensor data acquisition so as to monitor the equipment; (3) measuring the quality of Internet of Things equipment. In practice, the quality and efficiency of sensor data collection are always regarded to be an important factor to measure the quality of Internet of Things equipment.

The aim of this study was to introduce the dynamic change judgment model of sensor data and adjust the acquisition frequency in real time according to the change in sensor data. The change insensor data was calculated and judged according to data collected in the recent period, and the amount of data collected depended on the performance of the system. Feedback control was used to deal with the change in environment and make the model adaptive. We mainly investigated the acquisition timeinterval algorithm and the data changing degree judgment algorithm. 

The rest of this paper is organized as follows. Section 2 discusses the dynamic acquisition algorithm of sensor data, including judgment of the degree of data change, adjustment of acquisition time interval, dynamic acquisition model of sensor data, and the method of data fusion. Section 3 outlines the experiment, and Section 4 concludes the work.

## 2. Dynamic Acquisition and Fusion

### 2.1. Data Change Decision

In data change decision, we make decisions based on the data currently collected. It determines a collection window in advance and then fits the data in the collection window and predicts the data of the next collection point. After collecting the data of the next collection point, the predicted data and the practical data are compared. If the compared result exceeds the specified threshold, the data is regarded to be changing remarkably; otherwise, the data is considered to be changing slowly. 

During the whole movement process, due to the interference of external factors, the change in sensor data appears irregular. However, in a relatively small range, this change can be approximated by a function [14]. In order to facilitate calculation, the data in the acquisition window are processed by regression.

Suppose *m* to be the size of the collection window. For x_i_, which is one of the components of X, we continuously conduct the collection process *m* times, i.e., collect *m* times the values of x_i,1_, x_i,2_, …, x_i,m_ and then predict the value of x_i,m+1_ based on the collected data.

If the predicted value of x_i_ is x_i_’, then the absolute value of the degree of change will be |x_i_ – x_i_’|.

Let ${\bar{D}}_{i}^{m}$ denote the variance of *m* collection points related with x_i_. Then, |x_i_ – x_i_’|/${\bar{D}}_{i}^{m}$ denotes the relative changing degree related with x_i_. Obviously, the relative changing degree is a numeric number greater than 0, and in most cases, the number is less than 1. 

For the method of data change decision, we give a data changing degree threshold [ε^−^, ε^+^] in advance and then compare the value of actual change and the threshold. If the value of actual change is greater than the given threshold ε^+^, we regard the data change as exceeding the maximum linear errors permitted by the system, and the acquisition time intervals will be shortened to improve the fitting precision. If the actual change is less than the given threshold ε^−^, we regard the data change as lower than the minimum linear errors permitted by the system and then adopt the method that increases the acquisition timeintervals to decrease the bandwidth. If the actual change is within the interval [ε^−^, ε^+^], we regard the current acquisition timeintervals as better portraying the changing data, and the acquisition timeintervals are kept the same. Equation (1) formalizes these situations.

(1) $$\{\begin{array}{cc} |x_{i}-x_{i}\prime |/{\bar{D}}_{i}^{m}>\varepsilon^{+} & \mathrm{shorten}\;\mathrm{collecting}\;\mathrm{interval}\\ |x_{i}-x_{i}\prime |/{\bar{D}}_{i}^{m}<\varepsilon^{-} & \mathrm{increase}\;\mathrm{collecting}\;\mathrm{interval}\\ otherwise & \mathrm{mantain}\;\mathrm{collecting}\;\mathrm{interval} \end{array}$$

In Equation (1), x_i_ takes values according to the domain knowledge; x_i_’ is the predicted value of x_i_, whose unit is the same as x_i_; *m* is the size of the collection window, i.e., the number of samples; ${\bar{D}}_{i}^{m}$ is the variance of *m* samples; ε^−^ is the minimum threshold of data changing degree; ε^+^ is the maximum threshold of data changing degree. The size of the collection window, *m*, is set according to the domain, and the values of *m* are different in different fields. If *m* takes the larger values, more historical data is used for prediction and the predicting precision is higher, but the time consumption is larger. If *m* takes the smaller values, less historical data is used for prediction and the predicting precision is lower, but the time consumption is smaller. 

Equation (1) shows that the correction of the acquisition interval is not only related to the deviation between the current value and the mean value but also to the variance of the acquisition interval. This is an optimization process, and by constantly calculating deviations and variances, we can find a local optimal time interval.

### 2.2. Dynamic Regulation

When the system judges the data changing degree as greater than the preset threshold, it will adjust the acquisition timeintervals dynamically. In the following section, the dynamic regulation technique based on acquisition timeintervals is introduced.

**Definition** **1** (acquisition time slice, Δτ). The acquisition time slice is the basic unit of acquisition time, and the acquisition time interval consists of one or more acquisition time slices.

Suppose acquisition timeinterval to be T and T = KΔτ, where K takes the integers greater than or equal to 1. 

The basic unit of dynamic adjustment technology based on acquisition time interval is the time slice: When $|x_{i}-x_{i}\prime |/{\bar{D}}_{i}^{m}>\varepsilon^{+}$, the collection timeinterval will decrease Δτ, and this judgment will be conducted periodically, i.e., if $|x_{i}-x_{i}\prime |/{\bar{D}}_{i}^{m}>\varepsilon^{+}$ still holds, then the acquisition timeinterval will continue to decrease Δτ until the condition does not hold; when $|x_{i}-x_{i}\prime |/{\bar{D}}_{i}^{m}<\varepsilon^{-}$, the acquisition timeinterval will increase Δτ, and this judgment will be conducted periodically, i.e., if $|x_{i}-x_{i}\prime |/{\bar{D}}_{i}^{m}<\varepsilon^{-}$ still holds, the acquisition timeinterval will continue to increase Δτ; in other cases, the collection timeinterval will stay unchanged. 

**Figure 1:**
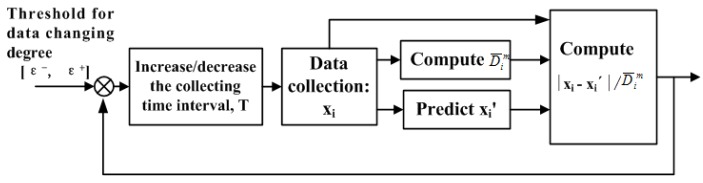
Dynamic regulation based on acquisition time and feedback control.

A dynamic regulation method based on acquisition time and feedback control is shown in Figure 1. 

In Figure 1, the control index is the data changing threshold [ε^−^, ε^+^], which is used to adjust the acquisition timeinterval; the controlled object is the collection timeinterval *T*, which is a variable; and the control law is the associated relationship formed by data collection, computing ${\bar{D}}_{i}^{m}$, predicting x_i_’, computing $|x_{i}-x_{i}\prime |/{\bar{D}}_{i}^{m}$, comparing [ε^−^, ε^+^], and so on. 

The implementation steps for dynamically regulating the acquisition timeinterval based on feedback control are as follows.

First step: Initialize system and define the size of the collection window, data changing degree threshold, and size of the acquisition time slice.
Second step: Start the timer and make the timeinterval of the timer equal to the acquisition timeinterval.
Third step: Conduct the data acquisition.
Fourth step: Collect the data in the acquisition window and compute the variance and predict the value of the next acquisition point.
Fifth step: Collect the next data point and conduct comparison with the predicted value and compute the relative changing degree.
Sixth step: Judge according to the changing degree, i.e., if the changing degree is larger than the given maximum threshold, decrease the current collection timeinterval; if the changing degree is smaller than the given minimum threshold, increase the current acquisition timeinterval.
Seventh step: Modify the current timeinterval of the timer and make it equal to the new acquisition timeinterval.
Eighth step: Execute steps 3–7 repeatedly until the end of data collection.

**Algorithm 1. Dynamic Adjustment Algorithm of Acquisition Time Interval**
**Input**: m, size of acquisition window; [ε^−^, ε^+^], threshold of data changing degree; T, acquisition timeinterval; Δτ, acquisition time slice; alpha, threshold of confidence; **Output**: S, acquisition dataset; 
**Design of algorithm:**
(1) Initialization (m, [ε^−^, ε^+^], Δτ); //System initialization (2) Start the timer with T;  //Start the timer with collection timeinterval T(3) while (acquisition is not terminated) { //data acquisition loop(4)   S ← conduct collection; (5)   W ← get data (S, m); //get the collect data to W(6)   Wvar = var(W);  //compute the variance of the dataset W(7)   x’←regress_predict(W, alpha); //predict by regress method(8)   x ← sampling(S); //collect the value of the next point(9)   if(|x - x’| / Wvar> ε^+^) {(10)    T = T – Δτ; //Decrease the acquisition timeinterval(11)   }else if(|x - x’| / War < ε^−^) {(12)    T = T + Δτ;  //Increase the acquisition timeinterval (13)   }(14) }  //The system converges or runs the prescribed iteration steps

**Remarks**: In Algorithm 1, firstly initialize the system according to the parameters, such as the size of acquisition window, data changing degree threshold, and acquisition time slice. Then, start the timer and set the initial collection timeinterval to be *T*. Finally, enter into the data acquisition loop. In the data acquisition loop, firstly save the collected data into the dataset *S*, then compute the variance of the data in the acquisition window according to the collected data, and then conduct the regression analysis for the data in the collection window and predict the value of the next acquisition point. According to Equation (1), judge whether the data changing degree exceeds the preset threshold. If the value of the relative changing degree is greater than the maximum data changing degree threshold, the acquisition timeinterval will be decreased by an acquisition time slice; if the value of the relative changing degree is less than the minimum data changing degree threshold, the acquisition timeinterval will be increased by an acquisition time slice; and in other circumstances, the acquisition timeinterval will be maintained. 

In addition, in the dynamic adjustment algorithm of acquisition timeinterval, the sampling interval is adjusted by adding or reducing a fixed time unit. When the deviation between the predicted value and the actual value is large, the increase or decrease of the calculation is linear and frequent. When the deviation between the predicted value and the actual value is small, the increase or decrease of the calculation will occur less. This is an effective adjustment method, which has three advantages: (1) simple and easy to implement; (2) high operating efficiency; (3) high fault tolerance if the time slice value is small.

The potential algorithms in this paper include data preprocessing, calculation of acquisition interval, acquisition of data transmission rate, dynamic adjustment of acquisition interval, etc. Their logical relationship is that data preprocessing is carried out first, and periodic cycle is then introduced. In the cycle, data are collected according to the current acquisition interval, and the data transmission rate is detected; the mean and variance are also computed at the same time. According to the transmission rate, adjust the acquisition interval or maintain the status quo so that the cycle continues until the completion of data acquisition.

Because this algorithm collects data from adjacent time intervals, the number of acquisitions is small, and the training can be completed in a very short time. In addition, there is a local correlation between the data, which also ensures the validity of the training model. The training model is a linear model. According to the current fitting model, the data of the next moment are predicted, and a new fitting model is then generated at the next moment to predict the data of the next time, i.e., iteration. 

Acquisition and fusion of sensor data is an organic whole. After the data of the first N frames are collected by several sensors, the average values are fused and compared with the actual values to determine the sampling rate of the next frame. 

## 3. Sensor Data Acquisition System

### 3.1. System Composition

**Figure 2:**
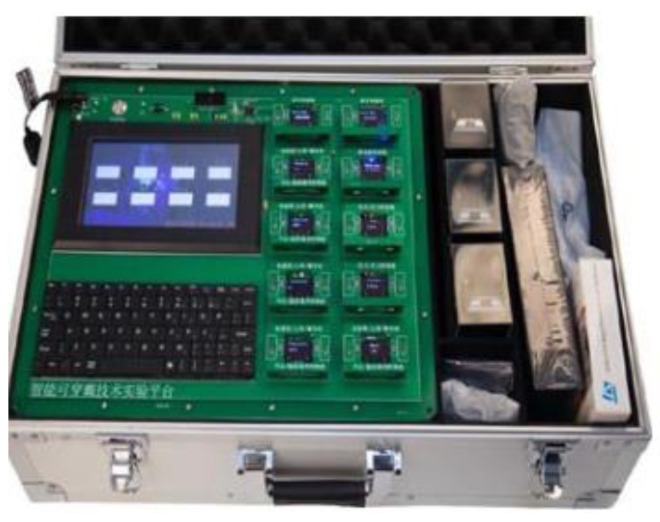
W5 experimental platform.

W5 is an experimental system for verifying sensor networks, which has the advantages of convenient operation, high accuracy, reliable transmission, and accurate positioning. W5 has been proven to be stable, functional, and expansible in multiparty testing and teaching, and it can play a good platform role in practical research. This training platform provides full ecological practice training for sensing equipment, which involves many contents, such as the Internet of Things technology, electronic engineering, computer engineering, mobile internet, communication engineering, biomedical electronics, and so on [18,19,20,21,22,23,24,25]. It is especially suitable for Internet of Things, electronic engineering, computer software, medical electronics, and communication engineering. An image of the system is presented in Figure 2.

**Figure 3:**
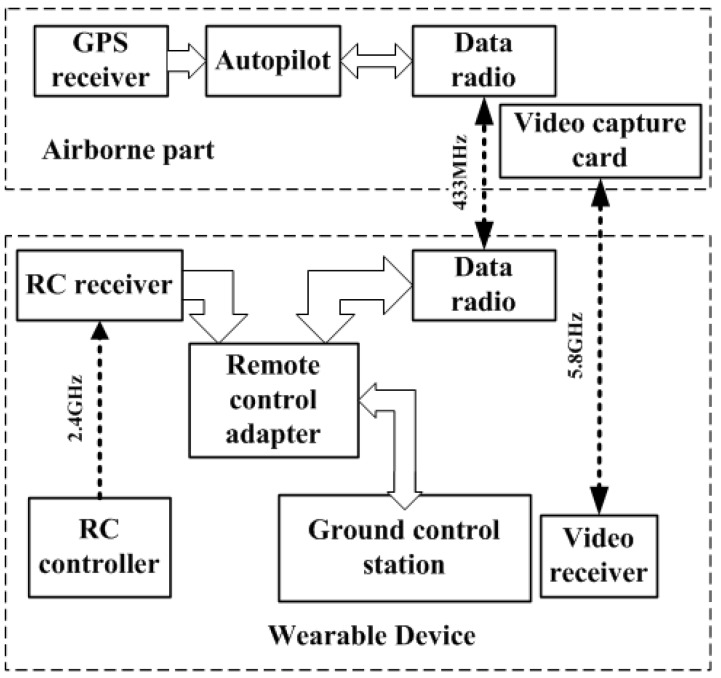
W5 sensor network.

The sensor network of W5 consists of ground control station, remote control adapter, radio control (RC) receiver, RC controller, data radio, autopilot, and so on, as shown in Figure 3. 

In Figure 3, the components of the W5 system are divided into two classes: sensor parts and controlled parts. The controlled parts include autopilot, airborne data radio, and so on, while the sensor parts include ground control station, RC receiver, RC controller, remote control adapter, ground data radio, and so on.

In the W5 system, there are three wireless transmission paths: the first is the wireless transmission path between RC controller and RC receiver, whose frequency is 2.4 GHz; the second is the wireless transmission path between ground data radio and airborne data radio, whose frequency is 433 MHz; and the third is the wireless transmission path between the video receiver on ground and the video capture card onboard, whose frequency is 5.8 GHz. 

There are three functional modules in the W5 system: (1) remote control module, i.e., the data link consisting of RC remote controller, RC receiver, remote control adapter, ground data radio, and airborne data radio, whose function is to provide the interfaces for users to remotely control the controlled objects; (2) planning and management module, i.e., the data link consisting of ground control station, remote control adapter, ground data radio, airborne data radio, and autopilot, whose function is route planning, task management, and automatic control; (3) video collection module, i.e., the data link consisting of the video receiver on the ground part and the video capture card on the airborne part, whose function is video collection, video display, and video recording. 

### 3.2. Sensing Data Acquisition 

The acquisition of sensor data involves different devices, including the following:

(1) Location identification: data obtained from the sensor of the global positioning system (GPS) receiver
(2) Number of satellites: data obtained from the sensor of the GPS receiver
(3) Control pattern: data obtained from the ground control station
(4) Type of reception: data obtained from the ground control station
(5) Identification of reception: data obtained from the sensor of the RC receiver
(6) Mark of automatic aerial photography: data obtained from the ground control station
(7) Mark of cycling route: data obtained from the ground control station
(8) Flight mode: data obtained from the sensor of the RC remote controller and the ground control station
(9) Types of taking off and landing: data obtained from the sensor of the RC remote controller and ground control station
(10) Longitude and latitude: data obtained from the sensor of the GPS receiver
(11) Heading: data obtained from the sensor of the autopilot
(12) Speed: data obtained from the sensor of the autopilot
(13) GPS altitude: data obtained from the sensor of the GPS receiver
(14) Barometric height: data obtained from the sensor of the autopilot
(15) Distance to the destination waypoint: data obtained from the sensor of the autopilot
(16) Lateral deviation distance: data obtained from the sensor of the autopilot

In order to facilitate research, the relevant variables are formalized, namely, location identification (x1), number of satellites (x2), control pattern (x3), type of reception (x4), identification of reception (x5), mark of automatic aerial photography (x6), mark of cycling route (x7), flight mode (x8), types of taking off and landing (x9), longitude (x10)and latitude (x11), heading speed (x12), GPS altitude (x13), barometric height (x14), distance to the destination waypoint (x15),and lateral deviation distance (x16):

X = [x1, x2, …, x16](2)

Obviously, X is a 16-dimensional vector. 

Vector is a good way to express input data. It expresses equations in a concise way, and it is suitable for multidimensional data applications. Therefore, the X represents the input data.

We can regard the sensor data as a time-varying function X(t) = f(t) = [x_1_(t), x_2_(t), … x_16_(t)]. Obviously, the changes in X(t) are related to the sensor device, and different time intervals correspond to different states. 

When the data changing degree is different, the acquisition period has a different influence on the change curve, i.e., the larger the acquisition period, the harder it is for the change curve to reflect the real situation; in contrast, the smaller the acquisition period, the greater the impact on the efficiency of the system. Therefore, the data acquisition algorithm based on equal timeinterval is low efficiency, which ignores the changing feature of acquisition data.

## 4. Experiments

**Figure 4:**
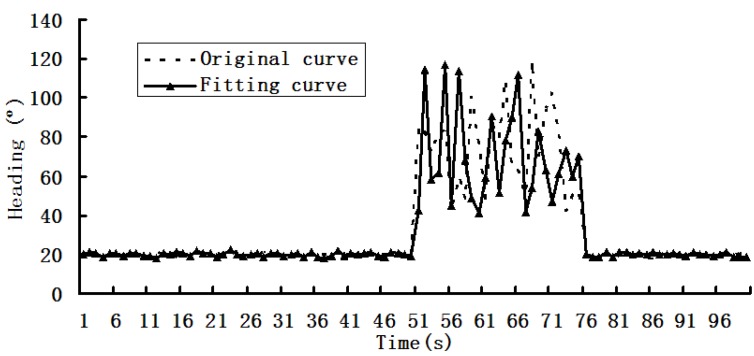
Changing situation of the sensing data.

**Figure 5:**
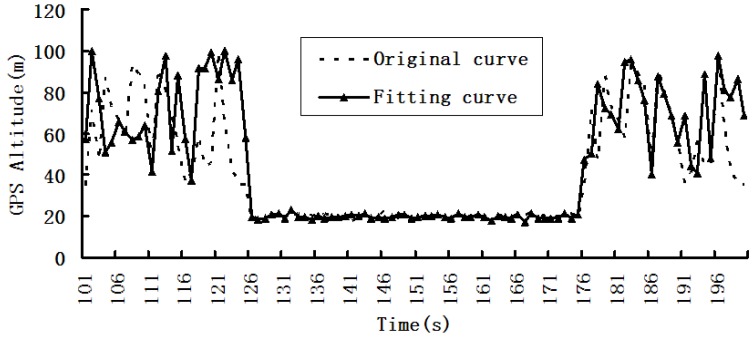
Changes in global position service (GPS) altitude in the 101–200 s time interval.

**Figure 6:**
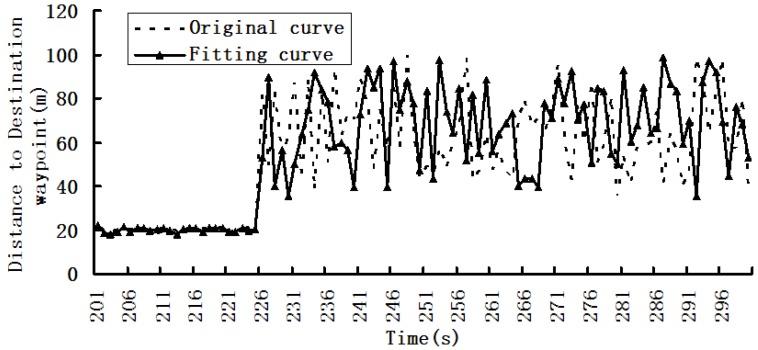
Distance to destination waypoint in the 201–300 s time interval.

We used W5 experimental device for dynamic data acquisition and fusion experiments, and the results are shown in Figure 4, Figure 5 and Figure 6.

Figure 4, Figure 5 and Figure 6 are the first phase of the experiment and show the changing situations of collection data in the following time intervals: 0–100, 101–200, and 201–300s. 

In Figure 4, it can be seen that the curve changes sharply in the 51–75 s time interval. If equal timeinterval collection is adopted, it will be hard to find the details of the changes. Therefore, within 51–75 s, the sampling rate needs to be reduced to find the detailed changes.

In Figure 5, the current sampling rate is too large for data collection in the 101–125 s and 175–200 s time intervals. Therefore, the sampling rate needs to be reduced to capture the data changes within the two time intervals. 

In Figure 6, the sampling rate between 201 and 225 s should be maintained, and the sampling rate in the 226–300 s time interval should be reduced. 

**Figure 7:**
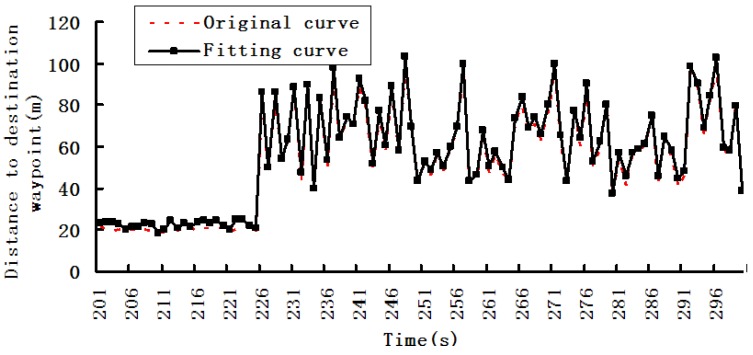
Result of dynamic regulation technique for the acquisition timeintervals.

The second phase of the experiment was environmental data collection. Here, the algorithm of dynamic regulation technique for the acquisition timeintervals was adopted, as shown in Figure 7. 

In Figure 7, according to the dynamic regulation technique, the collection effect in the 201–225 s time interval is basically the same as the changes in Figure 6.However, in the 226–300 s time interval, the collection effects has obvious enhancement, i.e., the collection effect is basically the same as the real changes and better than the results in Figure 6. 

In order to verify the effectiveness of this method, the data collected by the sensors were fused in stages. For the processing, the time estimation method [4], passive monitoring method [5], sampling method [6], and dynamic adaptive method were used, among which the first three are fixed acquisition intervals and the last one is dynamic acquisition interval.

Table 1 shows a comparison between the sensor data dynamic collection algorithm and the algorithms of [4,5,6]. 

In Table 1, “deviation to the desired value” denotes the standard deviation between the measured values and the desired values, i.e., Sqrt[(∑(x_i_ - x_i_’)^2) / (*m* – 1)]. In this formula, ∑ denotes the summation, x_i_’ denotes the desired value of x_i_, *m* denotes the size of the sampling window, and Sqrt denotes solving the square root. The desired values are measured by as little sampling timeinterval as possible. “Improvement (%)” means (deviation to the desired value in old method – deviation to the desired value in new method) / deviation to the desired value in new method * 100 %. 

**Figure 8:**
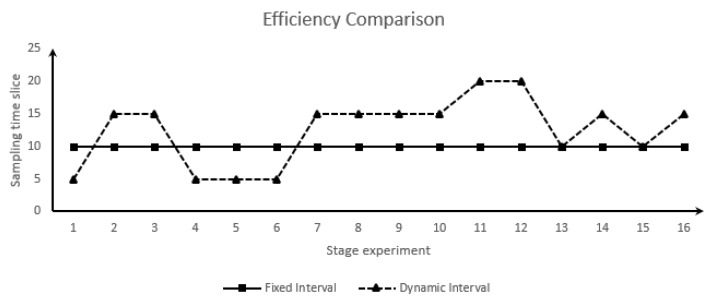
Efficiency comparison.

The efficiency of all stages of experiments was not significantly reduced under the premise of improving the experimental accuracy, as shown in Figure 8.

In Figure 8, the average sampling interval of the dynamic sampling method is 200/16=12.5, which is larger than that of the fixed sampling method. This reduces the processing time and data transmission time of the processor and improves the average efficiency.

The experimental effects (improvement) of the heading, distance to the destination waypoints, lateral deviation distance, and longitude and latitude experiments were the best, achieving over 300% improvement on average. The effects of the speed, GPS altitude, barometric height, and locating identification experiments were next, achieving about 20% improvement on average. 

## 5. Conclusions

Sensor data dynamic adaptive acquisition is a meaningful research topic. In this paper, an adaptive data acquisition method based on time interval dynamic adjustment is proposed. Sensor networks are located in a variety of complex natural environments, which makes it necessary for sensor data acquisition to adapt to them. The existing data processing methods based on sensor networks adopt fixed acquisition time interval, which not only wastes bandwidth but also reduces the accuracy of sensor data acquisition. By detecting and judging the degree of data change, the proposed method can dynamically adjust the time interval of sensor acquisition according to the data change, thus ensuring the efficiency and accuracy of acquisition. In this study, a wireless communication system with sensor devices was constructed to verify the effectiveness and efficiency of the proposed method.

## Figures and Tables

**Table 1:** Comparison between the collection algorithms.

Experimental Content	Fixed Acquisition Interval		Dynamic TimeInterval		Improvement (%)
	Sampling Interval (ms)	Deviation to the Desired Value	Sampling Interval (ms)	Deviation to the Desired Value	
**Heading experiment**	10	512.0	Dynamic	125.0	309.6
**Speed experiment**	10	51.4	Dynamic	41.2	24.8
**GPS altitude**	10	37.6	Dynamic	27.8	35.3
**Barometer height**	10	41.7	Dynamic	39.2	6.4
**Distance to waypoint**	10	682.7	Dynamic	122.8	455.9
**Lateral deviation Distance experiment**	10	75.4	Dynamic	23.4	222.2
**Locating identification**	10	450	Dynamic	180	150.0
**Number of satellites**	10	0	Dynamic	0	0
**Control mode**	10	0	Dynamic	0	0
**Reception types**	10	0	Dynamic	0	0
**Reception identification**	10	0	Dynamic	0	0
**Automatic photography**	10	0	Dynamic	0	0
**Cycling route identification**	10	0	Dynamic	0	0
**Flight pattern**	10	0	Dynamic	0	0
**Types of taking off and landing**	10	0	Dynamic	0	0
**Longitude and latitude**	10	5.8	Dynamic	1.2	383.3
